# Proteolytic Processing of ErbB4 in Breast Cancer

**DOI:** 10.1371/journal.pone.0039413

**Published:** 2012-06-22

**Authors:** Maija Hollmén, Ping Liu, Kari Kurppa, Hans Wildiers, Irene Reinvall, Thijs Vandorpe, Ann Smeets, Karen Deraedt, Tero Vahlberg, Heikki Joensuu, Daniel J. Leahy, Patrick Schöffski, Klaus Elenius

**Affiliations:** 1 Medicity Research Laboratories, Department of Medical Biochemistry and Genetics, University of Turku, Turku, Finland; 2 Turku Doctoral Programme of Biomedical Sciences, Turku, Finland; 3 Department of Biophysics & Biophysical Chemistry, Johns Hopkins University School of Medicine, Baltimore, Maryland, United States of America; 4 Department of General Medical Oncology, University Hospital Leuven, Leuven, Belgium; 5 Department of Surgery, University Hospital Leuven, Leuven, Belgium; 6 Department of Pathology, University Hospital Leuven, Leuven, Belgium; 7 Department of Statistics, University of Turku, Turku, Finland; 8 Department of Oncology, Helsinki University Hospital, Helsinki, Finland; 9 Leuven Cancer Institute, Leuven, Belgium; 10 Department of Oncology, Turku University Hospital, Turku, Finland; Ludwig-Maximilians University, Germany

## Abstract

ErbB4 is a receptor tyrosine kinase that can signal by a mechanism involving proteolytic release of intracellular and extracellular receptor fragments. Proteolysis-dependent signaling of ErbB4 has been proposed to be enhanced in breast cancer, mainly based on immunohistochemical localization of intracellular epitopes in the nuclei. To more directly address the processing of ErbB4 *in vivo*, an ELISA was developed to quantify cleaved ErbB4 ectodomain from serum samples. Analysis of 238 breast cancer patients demonstrated elevated quantities of ErbB4 ectodomain in the serum (≥40 ng/mL) in 21% of the patients, as compared to 0% of 30 healthy controls (*P* = 0.002). Significantly, the elevated serum ectodomain concentration did not correlate with the presence of nuclear ErbB4 immunoreactivity in matched breast cancer tissue samples. However, elevated serum ectodomain concentration was associated with the premenopausal status at diagnosis (*P* = 0.04), and estradiol enhanced ErbB4 cleavage *in vitro*. A 3.4 Å X-ray crystal structure of a complex of ErbB4 ectodomain and the Fab fragment of anti-ErbB4 mAb 1479 localized the binding site of mAb 1479 on ErbB4 to a region on subdomain IV encompassing the residues necessary for ErbB4 cleavage. mAb 1479 also significantly blocked ErbB4 cleavage in breast cancer cell xenografts *in vivo*, and the inhibition of cleavage was associated with suppression of xenograft growth. These data indicate that ErbB4 processing is enhanced in breast cancer tissue *in vivo*, and that ErbB4 cleavage can be stimulated by estradiol and targeted with mAb 1479.

## Introduction

ErbB4 (HER4) is a member of the ErbB subfamily of receptor tyrosine kinases (RTK) that also includes EGFR (also known as ErbB1 or HER1), ErbB2 (c-Neu, HER2) and ErbB3 (HER3). Similar to other RTKs, ligand interaction with ErbB4 promotes receptor dimerization, kinase activation, and initiation of phosphorylation-dependent signaling cascades, such as the mitogen-activated protein kinase (MAPK) and phosphoinositide 3-kinase (PI3-K)/Akt pathways [1], [2]. In addition to promoting these indirect signaling cascades, ErbB4 has been demonstrated to relay signals from the cell surface by releasing a soluble intracellular domain (ICD) fragment, in a process called regulated intramembrane proteolysis (RIP) [3], [4]. RIP involves shedding of the ErbB4 extracellular domain *via* the activity of tumor necrosis factor-alpha converting enzyme (TACE/ADAM17) [5] generating a substrate for a secondary cleavage at the plasma membrane by γ-secretase activity [4]. This produces the soluble ICD that can translocate into the nucleus where it functions as a transcriptional co-activator or co-repressor for a number of transcription factors, such as STAT5 [6], estrogen receptor (ER) [7], ETO2 [8], AP-2 [9], and TAB2-NCoR complex [10]. RIP, first discovered as a signaling mechanism for the Notch receptors [11] and the amyloid precursor protein [12], has during the last decade been shown to regulate signaling *via* an increasing number of cell surface proteins and RTKs in addition to ErbB4 [13].

An indication that RIP-mediated ErbB4 signaling may be biologically significant comes from the fact that the *ERBB4* gene is alternatively spliced creating isoforms (juxtamembrane isoform JM-a) capable of undergoing RIP, as well as isoforms (JM-b) that lack the TACE cleavage site [5], [14]. Biological relevance of RIP-mediated ErbB4 signaling has also been demonstrated *in vivo* in experiments indicating that soluble ErbB4 ICD regulates mammary epithelial development [15], and astrogenesis [10] in mouse. In addition, *in vitro* experimentation with transfected cell lines has demonstrated that the cleavable ErbB4 JM-a isoforms can promote profoundly different cellular responses as compared to the non-cleavable JM-b isoforms [9], [16]–[18]. Interestingly, different cancer tissues and cell lines seem to predominantly express cleavable ErbB4 isoforms [17], and down-regulation of ErbB4 expression in ER-positive breast cancer cells with ribozymes or siRNAs reduces growth [7], [19]. Furthermore, a monoclonal antibody mAb 1479, that specifically targets the cleavable ErbB4 JM-a isoform, has been shown to suppress breast cancer cell growth *in vitro* [20].

A subset of cancer tissues demonstrates nuclear ErbB4 immunoreactivty [21], [22], and nuclear ErbB4 immunoreactivity is associated with poor clinical outcome compared to membranous or cytoplasmic staining pattern in ErbB4-positive breast cancer [23], [24]. However, as the immunohistochemically detected nuclear ErbB4 staining pattern may not directly reflect the amount of ErbB4 cleavage, the extent of ErbB4 cleavage in tumor tissues *in vivo* has remained unclear. Here, we describe a novel ELISA method based on specific monoclonal ErbB4 antibodies for quantification of ErbB4 cleavage products in human serum samples. Using this ELISA we demonstrate that ErbB4 shedding is significantly enhanced in serum collected from patients diagnosed with early breast cancer compared to healthy individuals. We also demonstrate that a high serum ErbB4 concentration is associated with the premenopausal status, and that estradiol promotes ErbB4 cleavage and TACE activity *in vitro*. In addition, we address the effect of mAb 1479 on ErbB4 cleavage and breast tumor formation in a xenograft mouse model *in vivo*. Finally, we demonstrate by X-ray crystallography and mutagenesis studies that mAb 1479 binds to the C-terminal region of ErbB4 subdomain IV at a site encompassing JM-a-specific residues. These observations indicate that ErbB4 is cleaved in a subset of breast cancer tissues *in vivo*, and that mAbs targeted at ErbB4 may be a potential therapeutic option to suppress ErbB4 cleavage.

## Materials and Methods

### Ethics Statement

Written informed consent was obtained from all study participants. An Institutional Ethics Committee of the University Hospital Leuven approved the study. The study was performed according to the principles in the Declaration of Helsinki. All animal experiments were performed according to the Finnish Act on Animal Experimentation and international guidelines on animal welfare and approved by the National Animal Experiment Board.

### Serum and Tumor Tissue Samples

Paraffin-embedded tumor tissue samples were collected from 243 breast cancer patients who had undergone surgery at the University Hospital Leuven, Belgium in 2004 [25]. Serum samples collected before surgery were available from 238 (97.9%) of the patients. As a control, 30 serum samples were collected from healthy volunteers (24 females, 6 males) at the University of Turku, Turku, Finland.

### Sandwich ELISA for ErbB4 Ectodomain

Anti-ErbB4 ELISA based on two monoclonal antibodies, mAb 1482 and mAb 1532 recognizing different epitopes in the ErbB4 ectodomain [20], was set up and validated, as described in detail in File S1.

### ErbB4 Immunohistochemistry

Paraffin embedded sections (4 µm) were stained using a 1∶100 dilution of anti-ErbB4 HFR-1 (Neomarkers, Fremont, CA) recognizing the carboxy-terminal end of ErbB4, 1∶200 dilution of biotinylated anti-mouse secondary antibody, ABC kit, and diaminobenzidine (DAB) peroxidase substrate (all from Vector Laboratories, Burlingame, CA). The sections were counterstained with hematoxylin (Sigma-Aldrich, St Louis, MO). mAb 3g6 that recognizes a chicken T cell receptor served as a negative control, and Hermes-3 mAb (kindly provided by Dr. Sirpa Jalkanen, University of Turku, Turku, Finland) recognizing CD44, served as a positive control for each series of anti-ErbB4 stainings.

### Western Blot Analysis

To study the effect of estrogen on ErbB4 cleavage, MCF-7 human breast cancer cells (American Type Culture Collection (ATCC), Rockville, MD) were grown to 95% confluence in 6-well-plates and incubated for 24 hours in 500 µL of serum-free medium supplemented with or without 10 nM 17-β-estradiol or 100 ng/mL phorbol 13-myristate 12-acetate (PMA) (both from Sigma-Aldrich). The responsiveness of MCF-7 cells to 10 nM estradiol was controlled by MTS cell viability assays before experimentation. The amount of shed ectodomain was determined from 60 µL samples of conditioned medium by Western blotting with mAb 1479, as previously described [20]. To address the effects on the cellular ErbB4, cell lysates were analyzed by Western blotting with anti-ErbB4 (E-200) (Abcam, Cambridge, UK) and anti-actin (Santa Cruz Biotechnology, Santa Cruz, CA).

To study T-47D xenograft tumors, matching tissue samples from the harvested tumors were minced and lysed in lysis buffer on a rotator at +4°C for 30 minutes. Thereafter the protein content was analyzed by Western blotting using E-200 that recognizes the carboxy-terminal end of ErbB4, mAb 1464 [20] that recognizes the extracellular domain of ErbB4, or anti-actin as the primary antibodies. For blotting with mAb 1464, non-reducing conditions were used [20].

To analyze ErbB4 in T-47D cells *in vitro*, the cells were treated with or without 100 µM of the proteasomal inhibitor N-acetyl-L-leucyl-L-leucyl-L-norleucinal (ALLN) for three hours and analyzed by Western blotting with anti-ErbB4 (E-200).

To characterize the epitope for mAb 1479 binding, conditioned medium of COS-7 cells (ATCC) transiently expressing different His-tagged extracellular subdomains of ErbB4 was subjected to Western analysis with mAb 1479 and anti-pentaHis (Qiagen GmbH, Hilden Germany). For mAb 1479, non-reducing conditions were used [20].

### TACE Activity Assay

MCF-7 cells were cultured to 80% confluence on white flat-bottom 96-well plates (Perkin Elmer, Zaventem, Belgium). Cells were incubated for 30 min in PBS supplemented with or without 10 nM 17-β-estradiol or 100 ng/mL PMA. After incubation, the cells were washed and 100 µL of 10 µM TACE substrate peptide (Fluorescent substrate peptide II; R&D, Minneapolis, MN) in PBS was added. The generation of fluorescent end product was followed at 5 min intervals for 1 hour with a TECAN Ultra detector (Tecan Nordic AB, Mölndal, Sweden).

### Breast Cancer Cell Xenografts

A human breast cancer cell line, T-47D (ATCC) expressing endogenous ErbB4 JM-a was used to study the effect of mAb 1479 on tumor growth *in vivo*. Cells were suspended in a solution containing 50% RPMI and 50% Matrigel (BD Biosciences, San Jose, CA). Two million cells in a total volume of 50 µl were inoculated into the mammary fat pads of six-week old female SCID mice (Charles River, Sulzfeld, Germany). One day after inoculation the mice were treated with intraperitoneal injections of either 5 mg/kg of mAb 1479 or a control IgG antibody AK990/02 (Invivo Biotech Services, Berlin, Germany). The treatment was repeated every fourth day for three weeks. The mice were sacrificed three weeks after discontinuation of the treatments. As mAb 1479 is of the IgG2 isotype [20] with a half-life of approximately 3 weeks [26] the treatment was estimated to be effective throughout the duration of the experiment. The tumors were measured by a digital caliper and the tumor volume was calculated using the formula *T_vol_* = π/6 × larger diameter × (smaller diameter)^2^. Tumor lysates were subjected to Western analysis of ErbB4 expression.

### Plasmid Constructs and Transfection

To map the binding site of mAb 1479 in ErbB4, plasmids encoding each of the four subdomains (I, II, III, IV) of the extracellular domain (ECD) of ErbB4 coupled to carboxy-terminal His-tags were constructed as described in detail in File S1.

COS-7 cells plated on 6-well plates (1.5×10^5^) were transfected with 1 µg of the plasmids using FuGENE 6 transfection reagent (Roche, Mannheim, Germany).

### Crystallization and X-ray Data Collection

Purified 1479 Fab and the sErbB4:Fab1479 complex were crystallized and the structure determined as described in detail in File S1 and Table S1.

### Statistical Analyses

The cut-off for cancer-specific ErbB4 serum levels was searched by ROC curve analysis. Frequency tables were analyzed using either two-sided Chi-squared test or Fisher’s exact test. Non-normal distributions were compared using the Kruskal-Wallis test. Differences in the sizes of xenograft tumors were analyzed using the Mann-Whitney *U* test. Student’s *t*-test was used to compare densitometric analyses of the Western signals and effects on TACE activity. Both Mann-Whitney and Student’s *t*-test were two-sided and considered significant with the P-value <0.05.

## Results

### Nuclear ErbB4 Immunoreactivity in a Subset of Breast Cancer Samples

**Figure 1:**
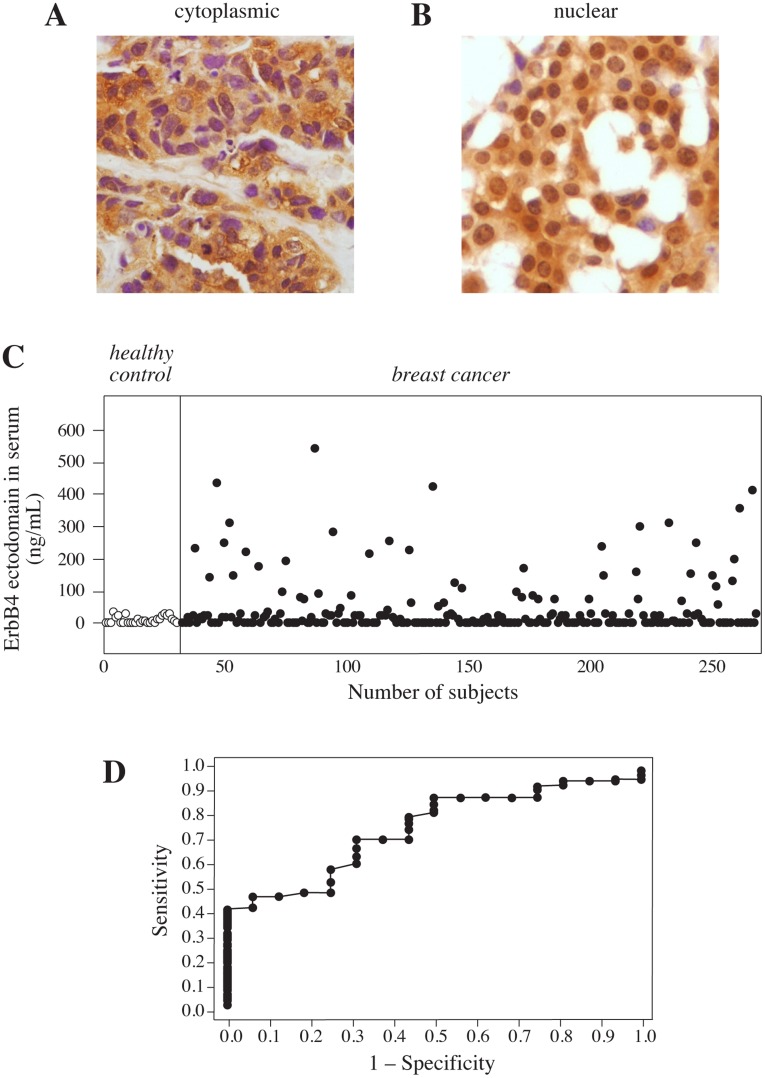
Immunohistochemical and ELISA analysis of ErbB4 expression in breast cancer tissues and matched serum samples. (A, B) Representative examples of immunohistochemical analysis of breast cancer sections with an antibody (HFR-1) recognizing the intracellular domain of ErbB4 demonstrate strong cytoplasmic immunoreactivity in the absence of nuclear staining (A), or strong cytoplasmic and strong nuclear immunoreactivity (B). (C) ELISA analysis of ErbB4 ectodomain levels in serum samples from 30 healthy individuals (white dots) and 238 breast cancer patients (black dots). (D) ROC analysis of the value of serum ErbB4 ectodomain concentration in differentiating between normal and cancer. AUC for ErbB4 concentration in serum was 0.76. At a cut-off of 40 ng/mL of ErbB4 ectodomain, the specificity of the assay was 100% and the sensitivity 43%. All samples with ErbB4 level below the detection limit of the assay (51% of cancer samples, 47% of control samples) were excluded from the analysis.

Localization of an intracellular epitope of ErbB4 in the nucleus may indirectly reflect the cleavage of ErbB4 at the cell surface, as cleavage is necessary for a soluble ICD to translocate into the nucleus [3]. To address the prevalence of ErbB4 immunoreactivity in the nucleus, paraffin-embedded tumor tissues were obtained from 243 patients diagnosed with early breast cancer at the University Hospital Leuven [25]. Tissue sections were stained with a monoclonal antibody (HFR-1) that recognizes the carboxy-terminal end of ErbB4. Both nuclear (Fig. 1A) and total (reflecting mainly immunosignal in the cytoplasm) (Fig. 1B) ErbB4 immunoreactivity was scored as either negative (0), weakly positive (1+), moderately positive (2+) or strongly positive (3+). In 3 cases the analysis was not interpretable or adequate tumor tissue was not available. Strong total ErbB4 expression was present in 44 (18%), moderate expression in 115 (48%), weak expression in 60 (25%), and no expression in 21 (9%) samples. Strong nuclear ErbB4 expression was present in 21 (9%), moderate in 37 (15%), weak in 67 (28%) cases, and no nuclear ErbB4 expression was detected in 115 (48%) tumors. All ErbB4-positive tumors showed cytosolic immunostaining, whereas none of the tumors had immunoreactivity solely in the nucleus.

Total ErbB4 expression in the present series was significantly associated with ER-positivity (*P* = 0.001; n = 235) and a high histological grade of differentiation (*P* = 0.004; n = 240) (Table 1). Nuclear ErbB4 signal was associated with total ErbB4 expression (*P* = 0.003; n = 240), and tended to be associated with ER-positivity (*P* = 0.09; n = 236), and high histological grade of differentiation (*P* = 0.07; n = 240) (Table 1). Frequency of cancer ErbB4 expression and subcellular localization of ErbB4 were similar in the current series to the breast cancer series studied previously using anti-ErbB4 immunohistochemistry [23], [24].

**Table 1:** Association of ErbB4 expression and localization with clinical variables.

	Total ErbB4 expression			ErbB4 in nucleus			ErbB4 in serum			All
	positive	negative		positive	negative		high	low		
	n (%)	n (%)	*P*-value	n (%)	n (%)	*P*-value	n (%)	n (%)	*P*-value	n (%)
Premenopausal	74 (34)	10 (48)		48 (38)	37 (32)		23 (48)	18 (29)		85 (35)
Postmenopausal	143 (66)	11 (52)	0.22	77 (62)	77 (68)	0.34	25 (52)	45 (71)	**0.04**	157 (65)
ER+	184 (86)	12 (57)		107 (87)	89 (79)		41 (87)	49 (78)		198 (83)
ER-	31 (14)	9 (43)	**0.001**	16 (13)	24 (21)	0.09	6 (13)	14 (22)	0.20	41 (17)
ErbB2+	31 (14)	1 (5)		18 (15)	13 (12)		8 (13)	4 (9)		32 (14)
ErbB2−	186 (86)	20 (95)	0.22	105 (85)	100 (88)	0.48	55 (87)	43 (91)	0.49	206 (86)
Grade 1	24 (11)	1 (5)		14 (11)	9 (8)		9 (14)	5 (10)		25 (10)
Grade 2	117 (53)	5 (24)		68 (54)	53 (46)		31 (49)	27 (55)		122 (50)
Grade 3	81 (36)	15 (71)	**0.004**	43 (35)	53 (46)	0.07	23 (37)	17 (35)	0.84	96 (40)
T1	66 (32)	5 (25)		44 (36)	27 (25)		17 (38)	20 (32)		72 (31)
T2	112 (53)	10 (50)		61 (50)	61 (57)		21 (47)	32 (52)		124 (53)
T3	32 (15)	5 (25)	0.51	18 (14)	19 (18)	0.22	7 (15)	10 (16)	0.83	37 (16)
N0	136 (62)	14 (67)		78 (62)	72 (63)		32 (65)	39 (62)		150 (62)
N≥1	83 (38)	7 (33)	0.68	48 (38)	43 (37)	0.91	17 (35)	24 (38)	0.71	90 (38)
M0	209 (95)	20 (95)		120 (96)	109 (95)		47 (96)	61 (97)		232 (95)
M1	10 (5)	1 (5)	1.0	5 (4)	6 (5)	1.0	2 (4)	2 (3)	1.0	11 (5)

*P* values <0.05 are shown in bold.

### ErbB4 Ectodomain Concentration in the Serum Associates with Premenopausal Status but not with Nuclear ErbB4 Immunoreactivity

To be able to more directly quantitate ErbB4 cleavage, a sandwich ELISA based on two specific monoclonal antibodies recognizing different epitopes on the extracellular domain of ErbB4 [20] was developed. ELISA was carried out for 238 serum samples collected from the same series that was also analyzed for ErbB4 immunostaining (Fig. 1C). One hundred and sixteen (49%) of the patients had detectable ErbB4 ectodomain serum levels ranging between 2 and 543 ng/mL. In the remaining 122 (51%) cases, the signal was below the detection limit of the assay. Serum samples obtained from a cohort of 30 healthy volunteers demonstrated ErbB4 ectodomain levels ranging between 0 and 38 ng/mL with a median of 7.8 ng/mL (Fig. 1C).

A cut-off between a normal and elevated serum ErbB4 level was determined using a ROC curve analysis where the best combination of specificity (100%) and sensitivity (43%) was obtained at 40 ng/mL (Fig. 1D). Using this cut-off, 50 (21%) out of the 238 breast cancer patients had an abnormally high ErbB4 ectodomain concentration in serum compared to none of 30 healthy controls (*P* = 0.002). When compared to the immunohistochemical analyses, high ErbB4 serum level among the cancer patients was not associated with either total (*P* = 0.50; n = 112) or nuclear (*P* = 0.94; n = 111) ErbB4 immunoreactivity. Elevated ErbB4 ectodomain concentration also did not associate with ER or ErbB2 expression, tumor grade or postsurgical stage (Table 1). However, a high ErbB4 serum level was significantly associated with the premenopausal status in the entire cohort (*P* = 0.04; n = 109), as well as in the ER-positive subgroup (*P* = 0.03; n = 90). There was no statistically significant association between high serum ErbB4 level and the premenopausal status in the subset of patients who had ER-negative cancer, but his subset was small in size (*P* = 1.00; n = 19). The small number of cancer recurrences (n = 3) or deaths (n = 5) during a follow-up period with a median duration of 5 years precluded studying the prognostic value of serum ErbB4.

These findings demonstrate that the amount of cleaved ErbB4 ectodomain can be quantitatively measured from clinical serum samples, and suggest that ErbB4 shedding is enhanced in a subset of human breast cancer. The data also indicate that immunohistochemical analysis of the subcellular ErbB4 distribution does not provide information about ErbB4 ectodomain shedding *in vivo*.

### Estradiol Enhances ErbB4 Shedding and TACE Activity

**Figure 2:**
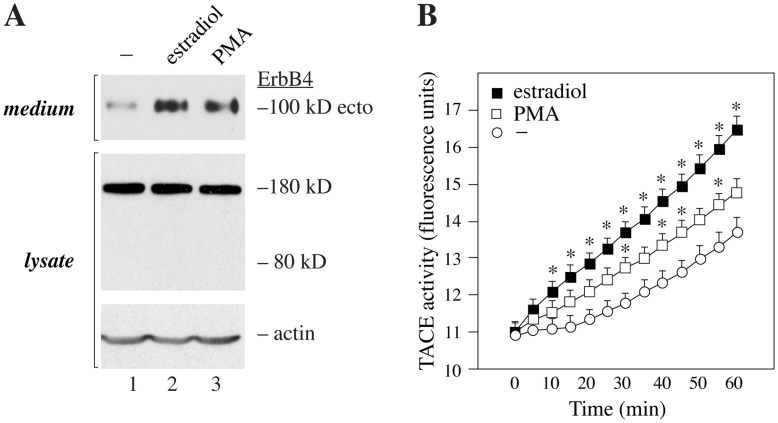
Effect of estrogen on ErbB4 shedding. (A) Western analysis of MCF-7 cells treated with or without 10 nM estradiol or 100 ng/mL PMA for 24 hours. The amount of shed ErbB4 ectodomain (100 kD) was detected with mAb 1479 under non-reducing conditions from culture medium and full-length ErbB4 (180 kD) with anti-ErbB4 (E-200) from cell lysates. Anti-actin was used as a loading control. The experiment was repeated four times with similar results. (B) TACE activity assay of MCF-7 cells treated for 30 min with or without 10 nM estradiol or 100 ng/mL PMA. After the treatments, TACE substrate peptide that becomes fluorescent upon cleavage was applied to the cells at time point 0. *, *P*<0.05 compared to control at the time point.

The observation that ErbB4 ectodomain shedding into the serum was more frequently detected in premenopausal patients with ER-positive cancer raised a hypothesis that estrogen signaling regulates ErbB4 shedding. To experimentally address this, estrogen-dependent ER-positive human MCF-7 breast cancer cells were treated for 24 hours with 10 nM estradiol and the amount of ErbB4 ectodomain accumulating in the conditioned medium was measured by Western blotting. Indeed, estradiol increased the amount of 100 kD ErbB4 ectodomain in the conditioned medium by 3.0 fold (Fig. 2A). For comparison, stimulation for 24 hours with the positive control 100 ng/mL PMA resulted in a 2.6 fold increase in ectodomain accumulation. Estradiol did not have a significant effect on the steady-state level of full-length 180 kD ErbB4 in the cell lysates (Fig. 2A).

Shedding of ErbB4 ectodomain has been shown to be regulated by the activity of TACE that cleaves ErbB4 within JM-a isoform-specific sequence at the extracellular subdomain IV [27]. To address whether estradiol promoted ErbB4 cleavage *via* TACE, TACE activity was measured from MCF-7 cells treated for 30 min with 10 nM estradiol using an assay based on a fluorescent substrate peptide. Estradiol enhanced TACE activity, and the effect was greater than the effect of 100 ng/mL of PMA (Fig. 2B), a known inducer of TACE activity [28]. These observations demonstrate that estrogen can stimulate ErbB4 ectodomain shedding, as well as activity of the ErbB4 sheddase TACE.

### mAb 1479 Binds ErbB4 at the Extracellular Subdomain IV

**Figure 3:**
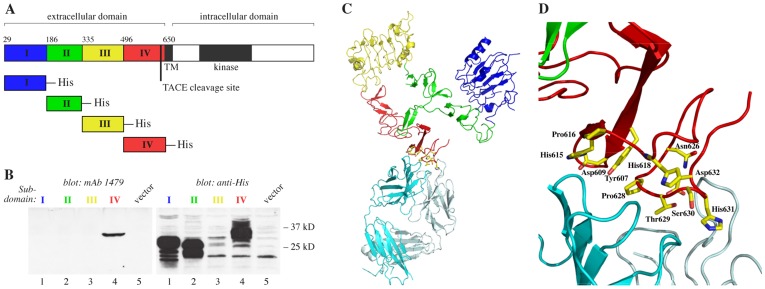
Mapping of the mAb 1479 epitope. (A) A schematic illustration of different His-tagged extracellular domain constructs generated to map mAb 1479 binding to ErbB4. (B) Western analysis of conditioned media from COS-7 cell transfectants expressing the different His-tagged subdomains of ErbB4 extracellular domain using mAb 1479 or anti-pentaHis as the primary antibody. (C, D) Crystal structure of the sErbB4:Fab 1479 complex. (C) A ribbon diagram of the sErbB4:1479 Fab complex is shown. sErbB4 is colored blue (subdomain I), green (subdomain II), yellow (subdomain III), and red (subdomain IV). The 1479 heavy chain is colored cyan and the light chain palecyan. The side chains of sErbB4 residues containing atoms within 4 Å of 1479 atoms are shown as stick models. (D) An expanded view of the sErbB4:Fab 1479 interface with sErbB4 residues containing atoms within 4 Å of Fab 1479 shown as stick models. The orientation is similar to that shown in panel C.

A monoclonal antibody, mAb 1479, that selectively binds the cleavable JM-a isoform of ErbB4, has been demonstrated to suppress ErbB4 cleavage *in vitro* [20]. The TACE cleavage site has been located between His651 and Ser652 in the extracellular subdomain IV of ErbB4 [27] within sequence that is only present in the JM-a isoform [14]. To map the binding site of mAb 1479 on ErbB4, His-tagged constructs encoding ErbB4 signal sequence followed by each of the four subdomains (I, II, III, or IV) of the ErbB4 extracellular domain were expressed in COS-7 cells (Fig. 3A), and analyzed by Western blotting with mAb 1479 as the primary antibody. mAb 1479 demonstrated specific interaction only with the subdomain IV (Fig. 3B). Lack of binding to the subdomain III was reproduced in independent experiments (data not shown). Interestingly, a point mutation H618P at subdomain IV abolished mAb 1479 binding to ErbB4 completely (Kurppa *et al*., unpublished data).

### X-ray Crystal Structure of the sErbB4:1479 Fab Complex

To further characterize the interaction between mAb 1479 and the extracellular domain of ErbB4, a 2.8 Å X-ray crystal structure of the 1479 Fab alone and a 3.4 Å crystal structure of the soluble ErbB4 ectodomain:1479 Fab complex were determined by molecular replacement using previously determined structures of sErbB4 [29] and related Fabs [30], [31] as search models (Fig. 3C). Although the moderate resolution of the complex structure precluded a detailed analysis, no major changes in either the Fab or sErbB4 structures were observed following complex formation. The 1479 Fab bound sErbB4 in a cleft between its heavy and light chains and contacted sErbB4 primarily on two loops (Tyr 607-His 618 and Asn 626-Asp 632) near the C-terminus of the ErbB4 extracellular region (Fig. 3C and D). Asn 626-Asp 632 are specific to the JM-a isoform of ErbB4, which nicely rationalizes the JM-a specificity of mAb 1479 [20]. Binding of 1479 near the C-terminal region of ErbB4 extracellular region is also consistent with its interference with proteolytic cleavage near this site.

### mAb 1479 Inhibits ErbB4 Cleavage and Tumor Growth in vivo

**Figure 4:**
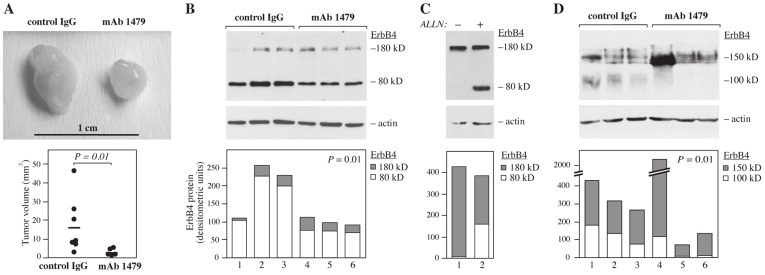
Effect of mAb 1479 on T-47D breast cancer cells. (A) Effect of mAb 1479 on human xenograft tumor growth. T-47D breast cancer cells were inoculated into mammary fat pads of female SCID mice. Mice were treated for three weeks with intraperitoneal injections of either anti-ErbB4 mAb 1479 (n = 5) or the negative control IgG (n = 7). Tumor volumes were measured six weeks after initiation of the treatments. Representative images of control IgG- and mAb 1479-treated tumors (top). mAb 1479 significantly (*P* = 0.01) reduced the mean tumor volume (bottom). Horizontal lines in the bottom panel indicate the mean tumor size. (B) Lysates from independent T-47D xenograft tumors treated with the control IgG (lanes 1–3) or mAb 1479 (lanes 4–6) were analyzed by Western blotting with an antibody recognizing the carboxy-terminus of ErbB4 (E-200) and with anti-actin as a loading control (top). Western signals for both 180 kD full-length ErbB4 (gray bars) as well as for the 80 kD carboxy-terminal fragment (white bars) were quantified by densitometry (bottom). The ratio of the 80 kD/(80 kD+180 kD) products reflecting the proportion of cleaved over total ErbB4 was significantly different between the two groups (*P* = 0.01). (C) Western analysis of ErbB4 expression in T-47D breast cancer cells *in vitro*. T-47D cells were treated with or without the proteasomal inhibitor ALLN (100 µM) for three hours and analyzed by Western blotting with anti-ErbB4 (E-200) (top). Western signals for both 180 kD full-length ErbB4 (gray bars) as well as for the 80 kD carboxy-terminal fragment (white bars) were quantified by densitometry (bottom). (D) The amount of shed ErbB4 ectodomain was directly analyzed from the same tumors as in B by Western blotting with an antibody against the extracellular domain of ErbB4 (mAb 1464) (top). Under non-reducing conditions, the full-length ErbB4 migrates at ?150 kD and ErbB4 ectodomain at ?100 kD [20]. Western signals for 150 kD full-length ErbB4 (gray bars) as well as for the 100 kD ectodomain (white bars) were quantified by densitometry (bottom). The ratio of the 100 kD/(100 kD+150 kD) products reflecting the proportion of shed over total ErbB4 was significantly different between the two groups (*P* = 0.01).

To further address the significance of ErbB4 cleavage *in vivo*, a mouse xenograft model was used. T-47D human breast cancer cells that naturally express the cleavable JM-a isoforms of ErbB4 (data not shown) were injected into the mammary fat pads of SCID mice and the mice were treated for three weeks with the JM-a isoform-specific anti-ErbB4 mAb 1479 or with a control IgG AK990/02 that does not recognize antigens in T-47D cells (data not shown). While tumors treated with the control antibody grew progressively and reached a median volume of 9.4 mm^3^ (range 3–47 mm^3^; n = 7), the mAb 1479-treated xenografts only reached a median volume of 1.9 mm^3^ (range 1.4–5.6 mm^3^; n = 5; *P* = 0.01) (Fig. 4A).

Tumors treated with the control antibody demonstrated significant basal accumulation of the carboxy-terminal 80 kD fragment as compared to the full-length 180 kD receptor, when analyzed by Western blotting with an antibody against the carboxy-terminal end of ErbB4 (E-200) (Fig. 4B, lanes 1–3). Consistent with inhibiting ErbB4 cleavage, treatment of the xenografts with mAb 1479 reduced the relative proportion of the 80 kD fragment of the total ErbB4 from an average of 90% to 74% (*P* = 0.01) (Fig. 4B, lanes 4–6).

Interestingly, when the same T-47D cells were cultured *in vitro*, the 80 kD fragment was barely detectable in the absence of proteasomal inhibition with 100 µM ALLN to prevent degradation (Fig. 4C). These data indicate that other factors, such as protein stability, in addition to ErbB4 cleavage by TACE, can significantly regulate the accumulation of the 80 kD fragment. Thus, to control the effect of mAb 1479 on ErbB4 cleavage *in vivo* using a read-out independent on factors regulating the carboxy-terminal 80 kD fragment, the amount of shed ErbB4 ectodomain was measured by Western analysis with an antibody (mAb 1464) against an extracellular ErbB4 epitope. The anti-ErbB4 mAb 1464 antibody only recognizes ErbB4 in its native conformation and requires non-reducing conditions where the soluble ectodomain migrates at 100 kD and full-length intact ErbB4 at 150 kD [20]. Again, mAb 1479 significantly reduced the relative proportion of the ectodomain fragment of total ErbB4 (from an average of 38% to 9%; *P* = 0.01) (Fig. 4D). Taken together, these findings indicate that the isoform-specific mAb 1479 can inhibit ErbB4 cleavage *in vivo* and that this inhibition is associated with suppression of xenograft tumor growth.

## Discussion

Recently, we demonstrated that a mouse monoclonal antibody mAb 1479 specifically recognizes the cleavable JM-a isoform of ErbB4 and suppresses growth of ER-positive breast cancer cells in both 2D and 3D conditions *in vitro* [20]. Since one of the proposed mechanisms underlying the anti-tumor effect of mAb 1479 is inhibition of ErbB4 JM-a cleavage and thus formation of a stable constitutively active receptor fragment [20], we addressed both its anti-tumor effect and effect on ErbB4 cleavage *in vivo*. A three-week treatment with mAb 1479 significantly reduced the volume of human ER-positive T-47D breast cancer cell xenografts in SCID mice. Consistent with the molecular mechanism involving ErbB4 cleavage our structural analyses demonstrated that the binding site of mAb 1479 on the ErbB4 ectodomain involved JM-a-specific residues 9 amino acids N-terminal to the previously characterized [27] cleavage site for the ErbB4 sheddase TACE. Furthermore, mAb 1479 therapy significantly inhibited ErbB4 cleavage in the xenograft model, as shown by Western analyses of both intra- and extracellular cleavage products. Immunological component in the observed anti-tumor mechanism of mAb 1479 was probably minor, as xenografts were analyzed in the immunocompromised SCID background, mAb 1479 suppresses T-47D growth also *in vitro* [20], and since mAb 1479 is of the IgG2 isotype [20] that only weakly interacts with a single FcγR (FcγRII) leading to inefficient ADCC or complement activation [26]. These data demonstrate that our previous findings about the effects of mAb 1479 on tumor growth and ErbB4 cleavage *in vitro* can be recapitulated in an experimental *in vivo* model.

Interestingly, while mAb 1479 blocks ErbB4 cleavage under both conditions, the proportions of the 80 kD carboxy-terminal ErbB4 fragment that basally accumulate *in vitro* and *in vivo* seem dramatically different. This was evident in our Western analyses of T-47D breast cancer cells grown either *in vitro* or as xenografts *in vivo*. *In vitro* less than 1% of the total ErbB4 was present as the 80 kD carboxy-terminal cleavage product, whereas in xenografts the percentage was 90%. No similar difference was found in the accumulation of ErbB4 ectodomain in breast cancer cell culture medium (data not shown) when compared to the ectodomain levels in the extracellular space [20] or serum of breast cancer patients. This indicates that a difference in the TACE-mediated cleavage of ErbB4 is not responsible for the observed difference in 80 kD fragment accumulation between *in vitro* and *in vivo* conditions. A more probable explanation is that the stability of the 80 kD fragment is reduced *in vitro* compared to *in vivo*, since treating the cultured breast cancer cells with a chemical inhibitor of proteasomal degradation strongly induced 80 kD fragment accumulation. It is also possible that mAb 1479-induced ectodomain clearing may selectively regulate ErbB4 ectodomain levels *in vivo*. These findings shed light on several clinical reports that have demonstrated the presence of carboxy-terminal epitopes of ErbB4 in the nuclei of a subset of breast cancer patients [23], [32], [33]. The specificity and relevance of the nuclear staining has been difficult to confirm, since under *in vitro* conditions, even with strongly overexpressed ErbB4 constructs, the nuclearly-localized carboxy-terminal fragment derived from a full-length ErbB4 has been barely detectable [3], [6], [16]. Taken together, these data suggest that accumulation of the cleaved carboxy-terminal ErbB4 fragment, but not ErbB4 cleavage itself, is significantly different between breast cancer cells *in vitro* and breast cancer tissue *in vivo*, and that the nuclearly localized ErbB4 epitope cannot be regarded as a surrogate marker of ErbB4 cleavage.

To establish a more reliable and direct biomarker for ErbB4 cleavage, an ELISA protocol based on two monoclonal antibodies, mAb 1536 and mAb 1482 [20], that recognize different epitopes in the ErbB4 ectodomain, was established and validated using recombinant ErbB4 ectodomain. The assay was sensitive for detection of ErbB4 ectodomain concentrations at the level of 1 ng/mL from human serum samples. Using this assay to test serum samples from the same series of patients that was characterized with ErbB4 immunohistochemistry we demonstrated that 50 (21%) out of 238 of patients had serum ErbB4 ectodomain levels over 40 ng/mL, the chosen cut-off level for an abnormally high ErbB4 ectodomain concentration. Thirty healthy volunteers had serum ErbB4 ectodomain levels ranging from 0 to 38 ng/mL, and none of these samples had a concentration exceeding the cut-off level (*P* = 0.002). Consistent with the predominant regulation of the 80 kD fragment at the level of protein stability, serum ErbB4 ectodomain level was not associated with nuclear or total ErbB4 immunoreactivity. These findings suggest that ErbB4 shedding does not reflect total tumor ErbB4 expression but rather the activity of ErbB4 sheddases, such as TACE, within the tumor. Consistent with this hypothesis, our *in vitro* experiments demonstrated that both estradiol and PMA stimulated ErbB4 shedding (probably by activating TACE) in the absence of significant changes in the steady-state levels of the cellular ErbB4 pool.

ErbB4 ectodomain measured from patient serum can potentially serve as a prognostic or predictive marker. The level of ErbB4 ectodomain was, however, not significantly associated with clinicopathological parameters such as ER status (*P* = 0.44) or histological grade of differentiation (*P* = 0.78). As overall immunohistochemical ErbB4 expression was associated with tumor ER-positivity (*P* = 0.001) and a high histological grade of differentiation (*P* = 0.004) in the same series, consistent with several previous reports [34]–[37], these findings imply that factors such as molecules promoting ErbB4 shedding may specifically interfere with the prognostic significance of serum ErbB4. One such molecule regulating ErbB4 shedding, identified here, was estradiol. It promoted ErbB4 shedding as well as TACE activity in ER-positive cancer cells *in vitro*. Moreover, a high concentration of shed ErbB4 ectodomain in the serum was significantly associated with the premenopausal status (*P* = 0.04), indicating that active endocrine estrogen signaling induced ErbB4 shedding also *in vivo*. Nevertheless, given the *in vitro* and *in vivo* observations that the anti-tumor effect of mAb 1479 was associated with its capability to suppress ErbB4 shedding, anti-ErbB4 ELISA is a promising predictive test for possible therapeutic applications of mAb 1479. In addition, our data imply that ER-positive breast cancer diagnosed in premenopausal women could be particularly sensitive to mAb 1479. Finally, combining mAb 1479 to tamoxifen might result in an additive effect in this subgroup as both compounds would be expected to suppress ErbB4 cleavage.

In conclusion, our findings indicate that regulated processing of ErbB4 is biologically relevant in breast cancer. In addition, we demonstrate that serum ErbB4 ectodomain concentrations can be measured from clinical samples with ELISA providing a potential novel bioassay for assessing ErbB signaling.

## Supporting Information

File S1 Supplemental materials and methods for biotinylation of mAb 1482, sandwich ELISA for ErbB4 ectodomain, plasmid constructs, preparation of the 1479 Fab and the sErbB4:1479 Fab complex, crystallization and X-ray data collection, and structure determination and refinement.
(DOC)

Table S1 Supplemental table showing X-ray data collection.
(DOC)

## References

[1] Kainulainen V, Sundvall M, Maatta JA, Santiestevan E, Klagsbrun M (2000). A natural ErbB4 isoform that does not activate phosphoinositide 3-kinase mediates proliferation but not survival or chemotaxis.. J Biol Chem.

[2] Sepp-Lorenzino L, Eberhard I, Ma Z, Cho C, Serve H (1996). Signal transduction pathways induced by heregulin in MDA-MB-453 breast cancer cells.. Oncogene.

[3] Ni CY, Murphy MP, Golde TE, Carpenter G (2001). gamma -Secretase cleavage and nuclear localization of ErbB-4 receptor tyrosine kinase.. Science.

[4] Lee HJ, Jung KM, Huang YZ, Bennett LB, Lee JS (2002). Presenilin-dependent gamma-secretase-like intramembrane cleavage of ErbB4.. J Biol Chem.

[5] Rio C, Buxbaum JD, Peschon JJ, Corfas G (2000). Tumor necrosis factor-alpha-converting enzyme is required for cleavage of erbB4/HER4.. J Biol Chem.

[6] Williams C, Allison J, Vidal G, Burow M, Beckman B (2004). The ERBB4/HER4 receptor tyrosine kinase regulates gene expression by functioning as a STAT5A nuclear chaperone.. J Cell Biol.

[7] Zhu Y, Sullivan LL, Nair SS, Williams CC, Pandey AK (2006). Coregulation of Estrogen Receptor by ERBB4/HER4 Establishes a Growth-Promoting Autocrine Signal in Breast Tumor Cells.. Cancer Res.

[8] Linggi B, Carpenter G (2006). ErbB-4 s80 intracellular domain abrogates ETO2-dependent transcriptional repression.. J Biol Chem.

[9] Sundvall M, Veikkolainen V, Kurppa K, Salah Z, Tvorogov D (2010). Cell death or survival promoted by alternative isoforms of ErbB4.. Mol Biol Cell.

[10] Sardi S, Murtie J, Koirala S, Patten B, Corfas G (2006). Presenilin-dependent ErbB4 nuclear signaling regulates the timing of astrogenesis in the developing brain.. Cell.

[11] Ray WJ, Yao M, Mumm J, Schroeter EH, Saftig P (1999). Cell surface presenilin-1 participates in the gamma-secretase-like proteolysis of Notch.. J Biol Chem.

[12] Gao Y, Pimplikar SW (2001). The gamma -secretase-cleaved C-terminal fragment of amyloid precursor protein mediates signaling to the nucleus.. Proc Natl Acad Sci U S A.

[13] McCarthy JV, Twomey C, Wujek P (2009). Presenilin-dependent regulated intramembrane proteolysis and gamma-secretase activity.. Cell Mol Life Sci.

[14] Elenius K, Corfas G, Paul S, Choi CJ, Rio C (1997). A novel juxtamembrane domain isoform of HER4/ErbB4. Isoform-specific tissue distribution and differential processing in response to phorbol ester.. J Biol Chem.

[15] Muraoka-Cook R, Sandahl M, Strunk K, Miraglia L, Husted C (2009). ErbB4 splice variants Cyt1 and Cyt2 differ by sixteen amino acids and exert opposing effects on the mammary epithelium in vivo.. Mol Cell Biol.

[16] Määttä JA, Sundvall M, Junttila TT, Peri L, Laine VJ (2006). Proteolytic cleavage and phosphorylation of a tumor-associated ErbB4 isoform promote ligand-independent survival and cancer cell growth.. Mol Biol Cell.

[17] Veikkolainen V, Vaparanta K, Halkilahti K, Iljin K, Sundvall M (2011). Function of ERBB4 is determined by alternative splicing.. Cell Cycle.

[18] Zeng F, Zhang M, Singh A, Zent R, Harris R (2007). ErbB4 isoforms selectively regulate growth factor induced Madin-Darby canine kidney cell tubulogenesis.. Mol Biol Cell.

[19] Tang CK, Concepcion XZ, Milan M, Gong X, Montgomery E (1999). Ribozyme-mediated down-regulation of ErbB-4 in estrogen receptor-positive breast cancer cells inhibits proliferation both in vitro and in vivo.. Cancer Res.

[20] Hollmén M, Määttä J, Bald L, Sliwkowski M, Elenius K (2009). Suppression of breast cancer cell growth by a monoclonal antibody targeting cleavable ErbB4 isoforms.. Oncogene.

[21] Srinivasan R, Gillett C, Barnes D, Gullick W (2000). Nuclear expression of the c-erbB-4/HER-4 growth factor receptor in invasive breast cancers.. Cancer Res.

[22] Hollmén M, Elenius K (2010). Potential of ErbB4 antibodies for cancer therapy.. Future Oncol.

[23] Junttila TT, Sundvall M, Lundin M, Lundin J, Tanner M (2005). Cleavable ErbB4 isoform in estrogen receptor-regulated growth of breast cancer cells.. Cancer Res.

[24] Thor A, Edgerton S, Jones F (2009). Subcellular localization of the HER4 intracellular domain, 4ICD, identifies distinct prognostic outcomes for breast cancer patients.. Am J Pathol.

[25] Brouckaert O, Pintens S, Van Belle V, Van Huffel S, Camerlynck E (2009). Short-term outcome of primary operated early breast cancer by hormone and HER-2 receptors.. Breast Cancer Res Treat.

[26] Salfeld J (2007). Isotype selection in antibody engineering.. Nat Biotechnol.

[27] Cheng QC, Tikhomirov O, Zhou W, Carpenter G (2003). Ectodomain cleavage of ErbB-4: characterization of the cleavage site and m80 fragment.. J Biol Chem.

[28] Doedens J, Mahimkar R, Black R (2003). TACE/ADAM-17 enzymatic activity is increased in response to cellular stimulation.. Biochem Biophys Res Commun.

[29] Bouyain S, Longo P, Li S, Ferguson K, Leahy D (2005). The extracellular region of ErbB4 adopts a tethered conformation in the absence of ligand.. Proc Natl Acad Sci U S A.

[30] Cho HS, Mason K, Ramyar KX, Stanley AM, Gabelli SB (2003). Structure of the extracellular region of HER2 alone and in complex with the Herceptin Fab.. Nature.

[31] Gardberg A, Dice L, Ou S, Rich R, Helmbrecht E (2007). Molecular basis for passive immunotherapy of Alzheimer’s disease.. Proc Natl Acad Sci U S A.

[32] Naresh A, Thor A, Edgerton S, Torkko K, Kumar R (2008). The HER4/4ICD estrogen receptor coactivator and BH3-only protein is an effector of tamoxifen-induced apoptosis.. Cancer Res.

[33] Srinivasan R, Gillett CE, Barnes DM, Gullick WJ (2000). Nuclear expression of the c-erbB-4/HER-4 growth factor receptor in invasive breast cancers.. Cancer Res.

[34] Bacus S, Chin D, Yarden Y, Zelnick C, Stern D (1996). Type 1 receptor tyrosine kinases are differentially phosphorylated in mammary carcinoma and differentially associated with steroid receptors.. Am J Pathol.

[35] Barnes N, Khavari S, Boland G, Cramer A, Knox W (2005). Absence of HER4 expression predicts recurrence of ductal carcinoma in situ of the breast.. Clin Cancer Res.

[36] Suo Z, Risberg B, Kalsson MG, Willman K, Tierens A (2002). EGFR family expression in breast carcinomas. c-erbB-2 and c-erbB-4 receptors have different effects on survival.. J Pathol.

[37] Sundvall M, Iljin K, Kilpinen S, Sara H, Kallioniemi O (2008). Role of ErbB4 in Breast Cancer.. J Mammary Gland Biol Neoplasia.

