# Supplementary material for: Proteolytic Processing of ErbB4 in Breast Cancer
Source: PLoS One. 2012 Jun 22;7(6):e39413. doi: 10.1371/journal.pone.0039413 (PMC3382207; doi:10.1371/journal.pone.0039413)
Supplement: File S1 — Supplemental materials and methods for biotinylation of mAb 1482, sandwich ELISA for ErbB4 ectodomain, plasmid constructs, preparation of the 1479 Fab and the sErbB4:1479 Fab complex, crystallization and X-ray data collection, and structure determination and refinement. (DOC) [file pone.0039413.s001.doc]

**Supplemental Materials and Methods**

**Biotinylation of mAb 1482.** For ELISA experiments, mAb 1482 that recognizes the ectodomain of ErbB4 [1] (diluted 1 mg/mL in PBS, pH 8.5) was biotinylated with 18% Biotin-NHS reagent (Calbiochem, Darmstadt, Germany) (dissolved 1 mg/mL in dimethyl sulfoxide) by a four-hour incubation at room temperature in a horizontal shaker. Unbound biotin-NHS was removed by dialysis.

**Sandwich ELISA for ErbB4 ectodomain.** Wells of white flat-bottom 96-well plates (Perkin Elmer, Zaventem, Belgium) were coated with 100 μL of mAb 1536 [1] (that recognizes a different epitope in ErbB4 ectodomain than mAb 1482) or with the negative control antibody 3g6 (kindly provided by Dr. Sirpa Jalkanen, University of Turku, Turku, Finland) at 10 μg/mL in 0.1 M NaHCO_3_ (pH 9.6) overnight at 4 °C followed by 1 hour at 37 °C. The wells were washed six times with a washing solution (0.1% Tween-20 in PBS) and then blocked to prevent non-specific absorption by adding 200 μL of a blocking solution (PBS containing 1% gelatin and 1% nonfat milk) for 45 minutes at room temperature. After washing the wells four times with the washing solution, 100 μL of each serum sample (diluted 1:25 in washing solution) was added into the wells followed by incubation for 1 hour at room temperature. The wells were washed again four times and incubated with 100 μL of the biotinylated mAb 1482 (10 μg/mL in washing solution) at room temperature for 1 hour. After four washes, 100 μL of streptavidin-horseradish peroxidase (GE Healthcare, Chalfont St Giles, UK) (diluted 1:1000 in washing solution) was added and the plates were incubated for 1 hour at room temperature. The plates were washed six times and finally developed for three minutes with ELISA POD chemiluminescence reagent according to the manufacturer’s instructions (Roche, Mannheim, Germany). The chemiluminescence signal was measured with TECAN Ultra.

Each assay included a dilution series of recombinant ErbB4 extracellular domain [1] that was used to generate a standard curve. To control for the protein milieu, the standard samples were diluted in the washing solution containing similar quantities of human serum (PromoCell GmbH, Heidelberg, Germany) as the patient samples. The human serum used for the standard samples did not contain detectable levels of ErbB4 ectodomain (data not shown). The mean signal obtained from the negative control wells (n = 4 in each assay; SD <15%) was subtracted from the mean signals of quadruplicate measurements with anti-ErbB4.

**Plasmid constructs.** To map the binding site of mAb 1479 in ErbB4, plasmids encoding each of the four subdomains (I, II, III, IV) of the extracellular domain (ECD) of ErbB4 coupled to carboxy-terminal His-tags were constructed using pcDNA3.1*ErbB4JM-aCYT-2* [2] as a template in PCR. The generated plasmids were named pcDNA3.1*ErbB4ECDI-His,* pcDNA3.1*ErbB4ECDII-His,* pcDNA3.1*ErbB4ECDIII-His* and pcDNA3.1*ErbB4ECDIV-His.* The sequence encoding the subdomain I was generated using a 5’-primer gaggcggccgcctgtgcaggaacggagaataaac and a 3’-primer ctacttaagttaatggtgatggtgatggtgtcctgaactaccatttgttgac, subdomain II using a 5’-primer gaggcggccgcctgtggacgttgccataagtcc and a 3’-primer ctacttaagttaatggtgatggtgatggtgacaagcttttgggcaaatgtcag, subdomain III using a 5’-primer gaggcggccgccgatggcattggcacaggatc and a 3’-primer ctacttaagttaatggtgatggtgatggtgattttcagcttttctgttgtcccg, and subdomain IV using a 5’-primer gaggcggccgcctgtactgctgaaggaatggtg and a 3’-primer ctacttaagttaatggtgatggtgatggtgtctagcatgttgtggtaaagtgg. The different subdomain-specific PCR products were inserted into a pcDNA3.1neo vector containing the ErbB4 signal sequence. A PCR product including ErbB4 signal sequence (25 amino acids) and part of the vector sequence was generated using a 5’ primer gggagatctcccgatcccctatgg and a 3’ primer gaggcggccgcagaatcgctgggctggacggtc from pcDNA3.1*ErbB4JM-aCYT-2* and inserted into pcDNA3.1neo before ligating the inserts encoding the subdomains.

**Preparation of the 1479 Fab and the sErbB4:1479 Fab complex**

Purified mAb 1479 supplied by Genentech, Inc. (South San Francisco, CA) (1 mg/mL) was incubated with 100 mM DTT at 37 °C for 1 hour and dialyzed into buffer containing 25 mM Tris-HCl (pH 7.5), 300 mM NaCl and 2 mM iodoacetamide for 48 hours at 4 °C. The reduced and alkylated IgG was then digested with Lys-C Endoproteinase (Roche) (1:1000 w/w). The digested proteins were loaded on a Protein A column (GE Healthcare) and Fab proteins were collected in the flow-through. The sErbB4:Fab 1479 complex was prepared by mixing the proteins with slight molar excess of sErbB4 followed by size-exclusion chromatography using a Superdex 200 26/60 column (GE Healthcare).

**Crystallization and X-ray data collection**

Purified 1479 Fab and the sErbB4:Fab1479 complex were dialyzed into buffer containing 2.5 mM Tris-HCl (pH 8.0) and 25 mM NaCl and concentrated to 7 mg/mL and 4 mg/mL, respectively. Both Fab 1479 and the sErbB4:Fab 1479 complex were crystallized by the hanging drop vapor diffusion method at 20 °C using 1 µL of protein and 1 µL of mother liquor. Fab 1479 was crystallized in 25% w/v polyethylene glycol 1000, 0.1 M sodium HEPES (pH 7.5). The sErbB4:Fab 1479 complex was crystallized in 10% w/v polyethylene glycol 4000, 0.2 M sodium acetate and 0.1 M sodium citrate (pH 5.5). Repeated macroseeding was performed for the sErbB4:Fab 1479 complex crystals to improve their diffraction quality. Before X-ray data collection, crystals were mounted in a nylon loop and cryo-protected in 16% (v/v) glycerol before flash cooling in liquid nitrogen. X-ray diffraction data for the sErbB4:Fab 1479 complex were collected at the GM/CA-CAT beam line of the Advanced Photon Source (APS) at Argonne National Laboratory (Argonne, IL) Diffraction data for Fab 1479 crystals were collected on a Saturn 944+ CCD detector using a Rigaku FR-E X-ray generator. X-ray data collection and refinement statistics of sErbB4:Fab 1479 and Fab 1479 crystals are shown in Suppl. Table 1.

**Structure determination and refinement**

The X-ray data were integrated using the HKL2000 program [3]. The structure of Fab 1479 was determined by molecular replacement with Phaser [4,5] A mouse IGg2a Fab structure (PDB code 2R0W) was used as the search model [6]. The sErbB4:Fab 1479 complex structure was determined by molecular replacement using programs MOLREP and Phaser [4,7]. The structures of sErbB4 (2AHX) and the Herceptin Fab (1N8Z) were used as search models [8,9]. Crystallographic refinement of the Fab 1479 structure was carried out using the programs REFMAC and autoBUSTER [10,11]. The sErbB4:Fab 1479 complex structure was refined using REFMAC, autoBUSTER and PHENIX [10-12]. The manual building of structures was performed using the graphics program COOT [13].

**References**

1. Hollmén M, Määttä J, Bald L, Sliwkowski M, Elenius K (2009) Suppression of breast cancer cell growth by a monoclonal antibody targeting cleavable ErbB4 isoforms. Oncogene 28: 1309-1319.

2. Määttä JA, Sundvall M, Junttila TT, Peri L, Laine VJ, et al. (2006) Proteolytic cleavage and phosphorylation of a tumor-associated ErbB4 isoform promote ligand-independent survival and cancer cell growth. Mol Biol Cell 17: 67-79.

3. Otwinowski Z, Minor W (1997) Processing of X-ray Diffraction Data Collected in Oscillation Mode; Carter JCW SR, editor. New York: Academic Press.

4. McCoy A, Grosse-Kunstleve R, Adams P, Winn M, Storoni L, et al. (2007) Phaser crystallographic software. J Appl Crystallogr 40: 658-674.

5. Potterton E, Briggs P, Turkenburg M, Dodson E (2003) A graphical user interface to the CCP4 program suite. Acta Crystallogr D Biol Crystallogr 59: 1131-1137.

6. Gardberg A, Dice L, Ou S, Rich R, Helmbrecht E, et al. (2007) Molecular basis for passive immunotherapy of Alzheimer's disease. Proc Natl Acad Sci U S A 104: 15659-15664.

7. Vagin A, Teplyakov A (1997) MOLREP: an automated program for molecular replacement. J Appl Cryst. pp. 1022–1025.

8. Bouyain S, Longo P, Li S, Ferguson K, Leahy D (2005) The extracellular region of ErbB4 adopts a tethered conformation in the absence of ligand. Proc Natl Acad Sci U S A 102: 15024-15029.

9. Cho HS, Mason K, Ramyar KX, Stanley AM, Gabelli SB, et al. (2003) Structure of the extracellular region of HER2 alone and in complex with the Herceptin Fab. Nature 421: 756-760.

10. Murshudov G, Vagin A, Dodson E (1997) Refinement of macromolecular structures by the maximum-likelihood method. Acta Crystallogr D Biol Crystallogr 53: 240-255.

11. Blanc E, Roversi P, Vonrhein C, Flensburg C, Lea S, et al. (2004) Refinement of severely incomplete structures with maximum likelihood in BUSTER-TNT. Acta Crystallogr D Biol Crystallogr 60: 2210-2221.

12. Adams P, Afonine P, Bunkóczi G, Chen V, Davis I, et al. (2010) PHENIX: a comprehensive Python-based system for macromolecular structure solution. Acta Crystallogr D Biol Crystallogr 66: 213-221.

13. Emsley P, Cowtan K (2004) Coot: model-building tools for molecular graphics. Acta Crystallogr D Biol Crystallogr 60: 2126-2132.
