# Supplementary material for: Proteolytic Processing of ErbB4 in Breast Cancer
Source: PLoS One. 2012 Jun 22;7(6):e39413. doi: 10.1371/journal.pone.0039413 (PMC3382207; doi:10.1371/journal.pone.0039413)
Supplement: Table S1 — Supplemental table showing X-ray data collection. (DOC) [file pone.0039413.s002.doc]

|  | Fab 1479 | sErbB4:Fab 1479 |
| --- | --- | --- |
| Data collection | | |
| Wavelength | 1.5418 | 1.0331 |
| Space group | P2_1_ | P2_1_2_1_2_1_ |
| Unit cell dimensions | | |
| a (Å) | 42.8 | 83.1 |
| b (Å) | 86.9 | 110.9 |
| c (Å) | 58.2 | 362.1 |
| Resolution (Å) | 2.5 | 3.4 |
| No. of unique reflections | 14679 | 44255 |
| Completeness (%) | 94.4 (86.0) | 95.2 (95.8) |
| I/σ | 9.5 | 6.2 |
| *R*_merge_ (%) | 13.6 (63.9) | 15.0 (61.2) |
| Redundancy | 2.9 (2.5) | 2.9 (2.3) |
| Refinement | | |
| Resolution (Å) | 25-2.5 | 45-3.4 |
| *R*_work_ (%) | 21.8 | 22.7 |
| *R*_free_ (%) | 26.6 | 26.7 |
| rmsd | | |
| Bond length (Å) | 0.007 | 0.009 |
| Bond angle (^o^) | 1.05 | 1.18 |

**Table S1**
